# Is Schizophrenia a Scapegoat? the Role of Childhood Traumas and Theory of Mind on Crime

**DOI:** 10.1002/cbm.70002

**Published:** 2025-07-19

**Authors:** Cetin Sahabettin, Kapubagli‐Cetin Nazlı, Sözeri‐Varma Gülfizar, Toker‐Ugurlu Tugce

**Affiliations:** ^1^ Department of Psychiatry Pamukkale University Faculty of Medicine Denizli Turkey; ^2^ Freelance Physician Denizli Turkey

## Abstract

**Background:**

Childhood traumas and low theory of mind abilities have been shown to be associated with violence, crime and schizophrenia. Understanding the factors that predispose to violent behaviour by patients with schizophrenia is important for treatment and safety.

**Aims:**

To investigate relationships between childhood trauma, theory of mind and violent behaviour in patients with schizophrenia and in a healthy comparison sample. Our hypothesis was that patients with schizophrenia who had been violent would be more likely to have a history of childhood trauma and show impairments in theory of mind impairments than either non‐violent patients with schizophrenia or healthy non‐violent people.

**Methods:**

In a cross‐sectional design, we recruited 30 patients with schizophrenia who had a history of violent crime, 50 patients with schizophrenia but no history of violence and 50 healthy people without history of violence. Each participant completed the positive and negative syndrome scale, childhood trauma questionnaire, the reading the mind in the eyes test and the hinting task.

**Results:**

Patients were significantly more likely to be male, without employment and on low income than healthy controls with a suggestion of the violent patient group being worst off. Childhood trauma history and theory of mind tests distinguished the schizophrenia groups from healthy controls but not from each other. Logistic regression analysis, comparing the schizophrenia groups only, confirmed that male sex and number of prior hospitalisations were the only two characteristics that independently distinguished the violent from the non‐violent groups with schizophrenia.

**Conclusions:**

Childhood trauma histories and theory of mind test results differed only between people with schizophrenia and healthy prosocial adults of similar age, but did not distinguish between the violent and non‐violent people with schizophrenia. Whereas a pathway to violence in the context of schizophrenia from early trauma through impaired reading of others' emotions seems plausible, it still lacks evidence. Our findings suggest good reason to assess and treat impairments of emotional perception and processing in people with schizophrenia, but that the need is unlikely to be specific to those who become violent. This needs further research with larger samples.

## 1 Introduction

Numerous studies have shown a higher rate of violence among people with schizophrenia or other psychoses compared to the general population (e.g., Soyka et al. 2007). In a systematic literature review, Fazel et al. (2009) reported the odds of violence among people with psychosis as about seven times that in the general population, although also noting that comorbid substance misuse accounted for much of the excess. Some of the risk factors for violence that apply within the general population also apply to people with schizophrenia, including male sex, young age, low socioeconomic status and low education level (Bo et al. 2011; Belli et al. 2010; Large and Nielssen 2011), but more understanding of the factors that predispose individuals with schizophrenia to violent behaviour and criminal activities is important for identifying patients with a tendency towards violence and offering them more effective treatments.

Exposure to adverse events, including trauma, in childhood has been shown to be significantly associated with violent behaviour in schizophrenia (Ranu et al. 2023). Oakley et al. (2016) reported a pathway from experience of trauma, through conduct disorder to violence in the context also of psychosis.

One factor that has been related to violence by people with schizophrenia is theory of mind impairment. A systematic review also showed a significant relationship between such social cognitive functions and childhood trauma in major psychiatric disorders such as schizophrenia (Rokita et al. 2018). By leading to impairments in theory of mind functions, early life trauma can negatively affect social skills generally, social problem solving specifically (Fett et al. 2011; Vaskinn et al. 2020, 2021). People with schizophrenia who have multiple childhood traumatic experiences use less effective communication styles (Spence et al. 2006).

It has been shown that, although patients with schizophrenia and a history of violence show better performance on some cognitive tasks than those without a history of violence, they struggle with empathic inferences (Abu‐Akel and Abushua'leh 2004). Engelstad et al. (2019) found that those with a history of homicide performed less well than patients with schizophrenia without a history of homicide in tasks requiring recognition of emotions from body language and in some theory of mind skills. In turn, impairments in social cognitive functions included in theory of mind are recognised as characteristic of schizophrenia (Green 2016).

The aim of this study is to investigate relationships between violent criminal behaviour by people with schizophrenia, history of childhood trauma and theory of mind. Our hypotheses were that patients with schizophrenia who have been violent will be more likely to report childhood trauma and show poorer performance on theory of mind tasks than non‐violent patients with schizophrenia but that both schizophrenia groups would perform less well on such tasks than healthy controls.

## 2 Methods

### 2.1 Ethics

Approval for the study was granted by the Tokat Provincial Health Department Ethics Committee (Decision Date: 13.09.2019, Meeting Number: 20, Decision Number: 06).

### 2.2 Participants

For this cross‐sectional study, we recruited patients from both inpatient and outpatient services of Tokat Dr. Cevdet Aykan Mental Health and Diseases Hospital between September 2019 and January 2020. This hospital serves an urban–rural mixed population of about one million. It is one of the few closed psychiatric services in the region, so patients are referred from various parts of the country but particularly from the Eastern Black Sea, Eastern Anatolia and Central Anatolia regions. Following initial assessments, 80 patients who met the inclusion criteria were enroled. Of these, 30 patients comprised the first group: individuals with a history of violent crime who had been found not criminally responsible due to their illness and were under mandated hospitalisation for protection and treatment. The second group included 50 patients with schizophrenia who had no history of criminal behaviour. Our third group of healthy, law‐abiding community controls as recruited from the hospital's staff and their relatives by using purposive sampling. Individuals were invited to participate if they matched the patient groups in age and were confirmed through psychiatric interview to have no current psychiatric disorder and no history of violent crime.

### 2.3 Inclusion and Exclusion Criteria

The offender‐patient group had to have a diagnosis of schizophrenia and have been deemed not responsible for a violent crime because of mental illness at the time as determined by a forensic psychiatric evaluation. According to Article 32 of the Turkish Penal Code, a person who cannot understand the legal meaning and consequences of the act they committed due to mental illness is not punished but ordered into to treatment in a high security hospital (Turkish Penal Code, Article 57).

The non‐violent patient group—inpatient or outpatient—had to have a diagnosis of schizophrenia but no history at all of physically violent behaviours.

The healthy participant group had to have no history of mental illness or physical violence.

Anyone with a history of loss of consciousness or neurocognitive impairment due to history of severe head trauma, developmental intellectual disability or other comorbid neuropsychiatric disorder was excluded. People whose psychotic symptoms were so severe as to be likely to interfere with testing were also excluded (greater than four points on at least one item of the PANNS).

### 2.4 Procedures

All consenting participants completed a sociodemographic and clinical data form and then the structured clinical interview for DSM‐5 (SCID‐5). The positive and negative syndrome scale (PANSS), the childhood trauma questionnaire, and tests to measure theory of mind abilities—the reading the mind in the ryes test (RMET) and the hinting task—were then administered, in that order.

### 2.5 Details of the Measures

#### 2.5.1 Sociodemographic and Clinical Data Form

This form was prepared by the researchers to record the sociodemographic characteristics of the participants, disorder and treatment history, personal and family history information and forensic history. See Supplementary Material for the full data form. To be able to compare patient medication, approximate chlorpromazine equivalent doses were calculated (Taylor et al. 2018).

#### 2.5.2 Positive and Negative Syndrome Scale (PANSS)

This semi‐structured interview scale consists of 30 items, each scored from one to seven points. There are 3 subscales of negative syndrome, positive syndrome and psychopathology. The scale was developed by Kay et al. 1987. Validity and reliability studies of the Turkish adaptation of the scale were conducted by Kostakoğlu et al. (Kostakoglu et al. 1999).

#### 2.5.3 Childhood Trauma Scale‐Short Form

This scale, developed by Bernstein and Fink (1998), consists of 28 items in 5 subgroups of childhood sexual abuse, physical abuse, emotional abuse, physical neglect and emotional neglect. The first form of 53 items was shortened by the original author and validity and reliability studies of this form in Turkish have been conducted (Sar et al. 2012). Each item is scored from 1 to five points and items including positive statements are reverse scored.

#### 2.5.4 Reading the Mind in the Eyes test

Participants are shown photographs of only the eye region of human faces and are asked to indicate from the presented descriptions the option that best explains the mental status of the person in the photograph (Baron‐Cohen et al. 2001). The original version of the test was composed of 36 items (Yıldırım et al. 2011). The internal consistency of four items was found to be low in the Turkish validity and reliability study, so these were removed from the Turkish version of the test. High points obtained in the test indicate that social cognition and theory of mind abilities are good (Yıldırım et al. 2011).

#### 2.5.5 Hinting Task

This cognitive theory of mind test, developed by Corcoran et al. (1995), evaluates the skill of being able to understand the underlying true intentions of statements expressed indirectly in communication between two people. The participant is asked what s/he thinks the person really wanted to say, and if the participant does not give the correct response to the first question, a second question is asked including a more clear intention. The test is scored as two points if the correct response is given to the first question, one point if the correct response is only given to the second question, and 0 points if neither response is correct. Only two of the 10 original stories from the test were translated into Turkish and used in the present study. These stories had previously been translated by three independent researchers for use in other research contexts (Ozguven et al. 2010; Yucel et al. 2016). The translations were then compiled into a single, unified version. To assess the face validity of the Turkish version, a back‐translation procedure was conducted, and the resulting evaluation confirmed that the content was appropriately preserved. During the translation process, necessary cultural adaptations were also made to ensure contextual relevance and linguistic clarity for the target population.

### 2.6 Statistical Analyses

Data were analysed statistically using SPSS 22.0 software (Statistical Package for the Social Sciences). In the comparisons of continuous data, parametric (Student's *t*‐test, ANOVA) or non‐parametric (Mann–Whitney *U* and Kruskal–Wallis) tests were used according to the distribution of the data, and the Bonferroni test was used in post hoc analyses. Categorical data were compared using the Chi‐squared test. Relationships between the results of the theory of mind tests and the childhood trauma questionnaire were evaluated with Spearman correlation analysis. Normality between independent variables was tested with the Kolmogorov–Smirnov test and Skewness and Kurtosis values.

#### 2.6.1 Logistic Regression Model

Logistic regression analysis was used to examine the independence of any relationships between the variables. The dependent variable was criminal violence history and the independent variables sex, education, income, living area, number of hospitalisations, antipsychotic drug dose (CPZ milligramme equivalents), childhood trauma questionnaire total score and reading the mind in the eyes test scores. The model was significant (omnibus test of model coefficients (*p* < 0.001), and the Nagelkerke *R* square value was used to assess the model's explanatory power. The model accounted for an overall percentage of 82.5; 47% of the variance in the dependent variable (*R* ^2^ adjusted = 0.476) was explained by the independent variables.

## 3 Results

### 3.1 General Characteristics of the Samples

The three groups were similar in age (37.80 ± 10.19 schizophrenia + violence group, 35.72 ± 11.19 non‐violent schizophrenia group and 35.24 ± 9.90 healthy non‐violent group; *p* = 0.555) and, as shown in Table 1, their usual living arrangements, albeit that none of the healthy non‐violent group was living in a patient care centre and a small minority of each patient group were doing so. Table 1 confirms that in all other aspects measured, there was something of a hierarchy, with the violent patient group at one extreme and the healthy non‐violent at the other. Thus the groups differed significantly by sex (only two women in the schizophrenia‐with‐violence group), work and income and whether rural or urban dwelling. Mean number of years of education also differed between the groups (7.16 ± 2.16 schizophrenia‐violence group, 8.28 ± 3.70 non‐violent schizophrenia group and 9.80 ± 4.80 for the healthy non‐violent group; (*p* = 0.012).

**TABLE 1:** Comparison of sociodemographic data between patients with schizophrenia who had been violent but found not criminally responsible for their index offence, non‐violent people with schizophrenia and healthy non‐violent controls.

		Schizophrenia with violent history (I)		Schizophrenia without violent history (II)		Healthy control (III)		*p* ^a^
		*n*	**%**	*n*	**%**	*n*	**%**	
Gender	Female	3	10.0	16	32.0	32	64.0	**< 0.001**
	Male	27	90.0	34	68.0	18	36.0	
Marital status	Married	8	26.7	9	18	34	68	**< 0.001**
	Single	22	73.3	41	82	16	32	
Working status	Active	6	20	12	24	43	86	**< 0.001**
	Student	0	0	5	10	4	8	
	Unemployed‐retired	24	80	33	66	3	6	
Living area	Rural	13	43.3	9	18	3	6	**< 0.001**
	Urban	17	56.7	41	82	47	94	
People lives with	Nuclear family	23	76.7	37	74.0	43	86.0	0.148
	Extended family	2	6.7	1	2	2	4	
	Alone	2	6.7	4	8	5	10	
	Patient care centre	3	10	8	16	0	0	
Income	Low	15	50	17	34	8	16	**0.001**
	Middle	15	50	28	56	27	54	
	High	0	0	5	10	15	30	

*Note:* Bold values indicate statistically significant ones.

^a^ Chi‐square test.

Table 2 confirms that the two patient groups were similar in terms of symptom presentation at the time of testing. They were also similar in terms of age of onset of illness, family history of illness, duration of illness without treatment, ever having had ECT, substance use and prior suicide‐related behaviour. The two groups did; however, differ on number of hospitalisations and on usual dose of antipsychotic medication. The violent group had had almost twice as many hospitalisations as the non‐violent group and medication doses were higher.

**TABLE 2:** Comparison of clinical characteristics and PANSS scores of with and without violent history groups.

		Schizophrenia with violent history (I)		Schizophrenia without violent history (II)		*p* ^a^
		Mean ± SD		Mean ± SD		
Illness onset age		25.33 ± 7.75		24.98 ± 8.30		0.851
Number of hospitalization		5.00 ± 3.58		2.40 ± 1.85		**0.001**
Antipsychotic drug dosage (milligram)#		644.16 ± 287.25		497.60 ± 257.55		**0.021**
Duration of illness without treatment (years)		2.10 ± 2.36		1.54 ± 1.64		0.334^b^
PANSS	Positive	14.96 ± 3.37		14.74 ± 3.71		0.785
	Negative	15.86 ± 3.72		16.16 ± 3.95		0.744
	Generally	35.06 ± 6.86		33.46 ± 6.72		0.308
	Total	65.90 ± 12.44		63.60 ± 12.23		0.421

		***n***	**%**	***n***	**%**	***p*** ^c^
Electroconvulsive treatment history	Yes	8	26.7	8	16.0	0.248
	No	22	73.3	42	84	
Comorbid medical disease	Yes	3	10	6	12	1.000
	No	27	90	44	88	
Lifetime substance use	Yes	6	20	3	6	0.073
	No	24	80	47	94	
Family history	Yes	4	13.3	6	12	1.000
	No	26	86.7	44	88	
Suicide history	Yes	8	26.7	9	18	0.359
	No	22	73.3	41	82	

*Note:* #Calculated over 100 mg/day chlorpromazine equivalent dose. Bold values indicate statistically significant ones.

Abbreviation: PANSS, positive and negative syndrome scale.

^a^ *t*‐test.

^b^ Mann‐Whitney *U* test.

^c^ Chi‐square test.

### 3.2 Histories of Abuse

Comparison of the groups by abuse history suggests similarities between all three groups in childhood experiences of emotional neglect and sexual abuse (Table 3) but the latter is probably accounted for by the very small number in each group reporting any sexual abuse. There were significant differences between the groups in reported emotional abuse, physical abuse, physical neglect and overall trauma scores, but mainly accounted for by the two schizophrenia groups differing from the healthy non‐violent comparison group. Only in respect of physical abuse was there any suggestion of difference between the two schizophrenia groups.

**TABLE 3:** Comparison of childhood trauma questionnaire and theory of mind group scores.

		Schizophrenia with violent history (I)	Schizophrenia without violent history (II)	Healthy control (III)	*p* ^a^	*p*1	*p*2	*p*3
Childhood trauma questionnaire	Emotional abuse	9.76 ± 3.21	9.32 ± 3.93	6.26 ± 1.49	**<** **0.001**	0.126	**<** **0.001**	**<** **0.001**
	Emotional neglect	10.53 ± 3.60	10.92 ± 4.90	9.06 ± 4.03	0.084^b^	1.000^b^	0.422^b^	0.097^b^
	Physical abuse	8.80 ± 3.22	6.82 ± 2.55	5.64 ± 1.95	**<** **0.001**	**0.002**	**<** **0.001**	**<** **0.001**
	Physical neglect	9.06 ± 2.81	8.12 ± 3.39	6.42 ± 1.70	**<** **0.001**	**0.032**	**<** **0.001**	**0.006**
	Sexual abuse	5.00 ± 0.00	5.66 ± 2.37	5.16 ± 0.86	0.112	0.050	0.174	0.273
	Total	43.00 ± 11.07	41.02 ± 15.03	32.48 ± 6.35	**<** **0.001**	0.064	**<** **0.001**	**<** **0.001**
Theory of mind	RMET	13.66 ± 3.98	16.06 ± 5.33	23.18 ± 3.71	**<** **0.001** ^b^	0.065^b^	**<** **0.001** ^b^	**<** **0.001** ^b^
	Hinting task	2.73 ± 1.22	3.08 ± 1.44	3.68 ± 0.55	**<** **0.001**	0.629	**<** **0.001**	**<** **0.001**

*Note:* p1: I–II, p2: I–III, p3: II–III. Bold values indicate statistically significant ones.

Abbreviation: RMET, reading the mind in the eyes test.

^a^ Kruskal‐Wallis Test.

^b^ ANOVA (Bonferroni test) p1, p2, p3 Mann‐Whitney *U* test.

### 3.3 Theory of Mind

The reading of the mind in the eyes test significantly differentiated the groups, with the schizophrenia with violence group having the lowest mean scores and the healthy controls the highest, the non‐violent people with schizophrenia occupied an intermediate position. There was, in fact, no significant difference between schizophrenia groups in this respect (Table 3).

The hinting task similarly differentiated the groups, but, again, there was no difference between schizophrenia groups.

Thus, all differences in theory of mind tests were essentially accounted for by differences between the combined schizophrenia groups and the healthy controls.

### 3.4 The Logistic Regression Model

The final logistic regression model, with violence as the dependent variable and only those variables that significantly distinguished the groups entered as independent variables is shown in Table 4 for the patient groups only. Only sex and number of hospitalisations distinguished the groups—with the odds of male sex being over nine times in the violent group and the number of hospitalisations.

**TABLE 4:** Logistic regression analysis with index violence (or not) as the dependent variable and each item significantly distinguishing the violent/not violent groups at binary level—data from the two schizophrenia groups only.

	OR	95% CI		*p*
Gender^a^	9.343	1.516	57.579	**0.016**
Education	0.934	0.715	1.220	0.615
Income^b^	0.948	0.272	3.311	0.934
Living area^c^	3.617	0.828	15.798	0.087
Number of hospitalization	1.489	1.125	1.971	**0.005**
Antipsychotic drug dosage (milligram)	1.001	0.998	1.003	0.630
Childhood trauma questionnaire total score	1.023	0.963	1.087	0.468
Reading the mind in the eyes test	0.891	0.770	1.031	0.120

*Note: Overall Percentage = 82.5*, *p* < 0.001, (*R* ^2^_adjusted_ = 0.476). Bold values indicate statistically significant ones.

Abbreviations: OR, odd's ratio; 95% CI, 95% confidence interval.

^a^ Male gender.

^b^ Low income.

^c^ Rural living.

## 4 Discussion

Understanding the factors that increase the risk of violence among people with schizophrenia is important for violence reduction among them. In this study, patients with schizophrenia who had committed violent crimes because of their disorder were compared with patients with schizophrenia but no criminal history and a healthy control group in respect of sociodemographics, clinical characteristics, childhood trauma history and some theory of mind functions. Childhood abuse histories and theory of mind functions significantly differed between schizophrenia groups and the healthy comparison group but did not differentiate between schizophrenia groups. Being male and having already had more admissions as an inpatient in a psychiatric hospital were linked to violence in the context of psychosis, but both psychosis groups had suffered adverse childhood events and struggled with theory of mind tasks. Malesness has long been recognised as a risk factor for violence among people with schizophrenia as well as more generally (e.g., Hastings and Hamberger 1997; Reichel 2017; Sturmey 2022). This may follow from social and cultural influences, such as gender roles and acceptance of male violence as normal, and patients with schizophrenia are not exempt from this. In a study in Turkey by Karabekiroğlu et al. (2016), it was reported that a group of people with schizophrenia and a history of violence were, on average, less educated and more likely to be unemployed than individuals with schizophrenia no history of violence; rural living was also associated with history of homicide. According to a previous review, the factors associated with violent behaviour in psychotic disorders included exposure to violence in adulthood, homelessness, male gender, non‐Caucasian ethnicity, low socioeconomic status, a history of childhood physical and sexual abuse and a parental history of crime or alcohol abuse (Witt et al. 2013).

### 4.1 Clinical Characteristics and Violence

At a binary level too, routinely observed clinical characteristics barely differntiated the schizophrmeia samples even at the binary level. Only antipsychotic dose levels and number of prior hospitalisations of all clinical characteristics measured differed significantly. These findings are probably indicative of more serious illness among the people who became violent, and here too there is consonance with the literature (e.g., Inan et al. 2018; Gurkan et al. 2019; Gumus et al. 2021 and Engelstad et al. 2019).

### 4.2 Childhood Trauma and Violence

In studies investigating the relationship between childhood trauma and violent behaviour in schizophrenia, a link has been established with childhood emotional abuse (Taskaynatan and Erol 2019), physical and sexual abuse (Witt et al. 2013), and exposure to domestic violence (Oakley et al. 2016). Del Pozzo et al. (2021) even suggested a link between such trauma and the degree of violence. This seems to fit with the ‘cycle of violence’ theory (Widom 1989), but an absence of difference in these respects in our sample cautions about simple assumptions about causal relationships.

One possible explanation for this finding is that childhood trauma may be highly prevalent among people with schizophrenia, regardless of whether they have a history of violence. This widespread exposure could have limited the emergence of statistically significant differences between the groups. Additionally, assessing trauma retrospectively through self‐report measures may have introduced recall bias, particularly in relation to emotional forms of abuse. Indeed, in our study, the pre‐regression group comparisons revealed that histories of physical neglect and physical abuse were significantly higher among those with a history of violence.

### 4.3 Theory of Mind and Violence

Misinterpretation of social cues can lead to less socially appropriate behaviours including violence (Weiss et al. 2006). Difficulties in mentalisation (Bo et al. 2014), lack of empathy (Abu‐Akel and Abushua'leh 2004), problems in recognising emotions from body language and facial expressions and impairments in both affective and cognitive components of theory of mind (Engelstad et al. 2019; Vaskinn et al. 2023, 2024) have all been associated with violent behaviour. However, the relationship between impaired theory of mind and violence has not been consistently demonstrated across studies. A meta‐analysis by Sedgwick et al. (2017) underlined that the evidence for this association remains inconclusive. Some studies have shown that patients with and without a history of violence do not differ significantly in their ability to recognise emotions from facial expressions or to infer intentions, and that patients with a history of violence may even be better at identifying negative emotions such as fear and disgust Demirbuga et al. 2013; Engelstad et al. 2019; Iozzino et al. 2021).

In these studies, social cognitive functions have been assessed using a range of tools including photographs, images, videos and narratives. Social cognition encompasses more than just emotion recognition; it includes more complex processes such as understanding others' beliefs, intentions and thoughts—that is, the theory of mind. The diversity of measurement tools may partly account for the inconsistencies observed across findings. In our study, we used the reading the mind in the eyes test (RMET) to assess the affective component and the hinting task to evaluate the cognitive component of theory of mind. Whereas patient groups performed significantly worse than healthy controls, they did not differ significantly from one another.

The lack of difference in this respect between the schizophrenia groups may be more reflective of their stage or type of treatment than of personal differences. In our sample, patients who had committed violent offences were subjected to forensic psychiatric assessment and, if deemed not criminally responsible, were transferred to specialised institutions. These processes are carried out under the close and regular supervision of judicial authorities. Consequently, such patients typically have greater access to both inpatient and outpatient care, regular follow‐up, pharmacological treatment and psychosocial rehabilitation. The significantly higher number of hospitalisations observed in the violent schizophrenia group in our study may reflect this dynamic. It is possible that consistent treatment and structured support have contributed to relatively preserved social cognitive performance in this group.

### 4.4 Limitations

There were several limitations to this study, primarily that causal relationships between the findings could not be established because of the cross‐sectional design. Evaluating childhood trauma in adults with a scale requiring recall of long past experiences, is likely to decrease the reliability and other qualities of the data, but not to enquire about past traumas at all would leave an even bigger gap.

Another problem for this research was the rather small sample size. In addition, variability in the time elapsed between the violent offence and the research assessment may have influenced the results. For the majority of cases, it was not possible to obtain clinical data from the period during which the violent act occurred. In practice, individuals who commit a violent offence may be referred for psychiatric evaluation at any stage of legal proceedings if the presiding judge deems it necessary. In such cases, the individual is referred to a psychiatric clinic for an expert opinion. If the forensic psychiatric committee concludes that the individual lacked criminal responsibility at the time of the offence, they are transferred to a secure hospital facility. As this process varies considerably from case to case, the time interval between the offence and participation in the study is not consistent. Moreover, the theory of mind tests used in our research are not part of routine forensic psychiatric assessments.

Another limitation is that healthy controls were matched for age but not for gender. While healthy controls were matched by age, they were not matched by gender. For example, both the original development study of the reading the mind in the eyes test (Baron‐Cohen et al. 2001) and its Turkish validation (Yıldırım et al. 2011) have shown that female participants tend to score significantly higher than males. In our study, participants' legal histories were obtained through self‐report. Finally, the degree of violence involved in the offence committed by each patient was not assessed.

## 5 Conclusion

In this study, we examined individuals who had engaged in violent behaviour either directly or indirectly related to their psychiatric disorder. With regard to the study's focus on childhood trauma and theory of mind tasks, our initial hypotheses were not confirmed. Although histories of physical abuse and neglect were found to be significantly higher in the group of patients with schizophrenia and a history of violence, these differences lost their statistical significance in the regression analysis. Moreover, trauma and theory of mind measures did not predict violent behaviour within the patient groups. These findings serve as a reminder that simplistic and reductionist explanations of violent behaviour should be avoided. Violence is the result of a complex interacting factors.

There are few studies in the literature that have jointly assessed both affective and cognitive components of theory of mind in conjunction with childhood trauma. From this perspective, our study addresses an important gap. Although our hypotheses were not supported, the multidimensional nature of the topic and the diversity of measurement tools used indicate a clear need for further research using larger samples and varied methodologies.

It is essential not to reduce violence solely to psychiatric diagnosis but instead to focus on broader modifiable factors. As Bo et al. (2014) have pointed out, the majority of individuals diagnosed with schizophrenia do not exhibit violent behaviour, and the diagnosis itself is of limited value in assessing violence risk. In this context, the childhood trauma histories and theory of mind abilities explored in our study may be considered as potentially modifiable domains. Del Pozzo et al. (2021) emphasise that early identification of childhood trauma following a first psychotic episode, together with timely interventions, could be effective in reducing violent behaviour. Similarly, Jones et al. (2019) demonstrated that social cognitive training may reduce violence in individuals with severe psychiatric disorders. It is also important to recognise that risk factors commonly associated with violent behaviour in the general population—such as low socioeconomic status, male gender, substance use and a family history of violence—are also relevant in schizophrenia. Identifying such preventable and modifiable risk factors is not only important for the prevention of violence, but also for combatting the stigma that associates schizophrenia with violence. Psychosocial rehabilitation programmes targeting trauma and social cognition, along with structural changes at the level of social policy, could offer constructive contributions to this multifaceted problem. Nevertheless, there remains a need for further multi‐centre studies evaluating both trauma and theory of mind using a variety of assessment tools.

## Conflicts of Interest

The authors declare no conflicts of interest.

## Supporting information

Supplementary Material

## Data Availability

The data that support the findings of this study are available from the corresponding author upon reasonable request. Because of privacy or ethical restrictions, the raw data are not publicly available.
