# Supplementary material for: Is Schizophrenia a Scapegoat? the Role of Childhood Traumas and Theory of Mind on Crime
Source: Crim Behav Ment Health. 2025 Jul 19;35(4):187–96. doi: 10.1002/cbm.70002 (PMC12374778; doi:10.1002/cbm.70002)
Supplement: Supplementary file 1 — Supplementary Material [file CBM-35-187-s001.docx]

**SOSYODEMOGRAFİK VE KLİNİK VERİ FORMU**

**Yaş: Cinsiyet:**

**Medeni durum:** evli – bekar – dul – boşanmış

**Çocuk:** yok – var (sayı:….)

**Kardeş sayısı:**

**Yaşadığı yer:** kırsal / kentsel **Birlikte yaşadığı kişiler:**

**Eğitim düzeyi (yıl):**

okur yazar değil – okur yazar – ilköğretim – lise - üniversite

**Meslek:**

çalışıyor – öğrenci – emekli - çalışmıyor

**Gelir durumu:**

**Geçirilmiş/ mevcut psikiyatrik hastalık öyküsü:**

**İlk psikiyatri başvuru yaşı: Hastanede yatış sayısı:**

**Hastalık süresi: Tedavisiz geçen hastalık süresi:**

**Poliklinik kontrol düzeni:**

**Halen devam eden tedavi (süresi ve günlük doz): EKT öyküsü:**

**Ek tıbbi hastalık: Alkol, madde ve sigara kullanımı:**

**Birinci derece yakınında psikiyatrik hastalık öyküsü:**

**Kendine zarar verme/ intihar girişimi:**

**Geçmişte yaşanan önemli olaylar (Göç, Erken yaş ebeveyn kaybı…)**

**Adli öyküsü (suçun niteliği, varsa TCK 32. Madde kararı):**

**SOCIODEMOGRAPHIC AND CLINICAL DATA FORM**

**Age:**                 **Gender:**

**Marital status:** married – single – widowed – divorced

**Children:** none – yes (number: ___)

**Number of siblings:**

**Place of residence:** rural / urban  **People living with:**

**Educational level (years):**
illiterate – literate – primary school – high school – university

**Occupation:**
employed – student – retired – unemployed

**Income level:**

**History of past/current psychiatric illness:**

**Age at first psychiatric consultation:**  **Number of psychiatric hospitalizations:**

**Duration of illness:**      **Untreated duration of illness:**

**Outpatient follow-up pattern:**

**Current treatment (duration and daily dosage):**  **History of ECT:**

**Comorbid medical conditions:**  **Use of alcohol, substances, and tobacco:**

**Family history of psychiatric illness (first-degree relatives):**

**History of self-harm/suicide attempts:**

**Significant past life events (e.g., migration, early parental loss):**

**Forensic history (nature of offense, presence of Article 32 of the Turkish Penal Code if applicable):**
